# Capillaroscopic findings in Turkish Takayasu arteritis patients 

**DOI:** 10.3906/sag-1812-223

**Published:** 2019-10-24

**Authors:** Ali AKDOĞAN, Abdulsamet ERDEN, Esra FIRAT ŞENTÜRK, Levent KILIÇ, Alper SARI, Berkan ARMAĞAN, Ömer KARADAĞ, Sedat KİRAZ

**Affiliations:** 1 Department of Internal Medicine, Division of Rheumatology, Faculty of Medicine, Hacettepe University, Ankara Turkey; 2 Department of Internal Medicine, Faculty of Medicine, Hacettepe University, Ankara Turkey

**Keywords:** Takayasu arteritis, vasculitis, capillaroscopy, vasculitic syndromes, abnormality

## Abstract

**Background/aim:**

Abnormal capillaroscopic findings have been reported in vasculitic syndromes such as Behçet’s disease, Henoch–Schönlein purpura, and Wegener’s granulomatosis. This study was conducted to define the capillaroscopic changes in patients with Takayasu arteritis (TA).

**Materials and methods:**

We studied 28 TA patients (27 females). The nail folds from the 2nd to 5th fingers on both hands were examined with video capillaroscopy for all. A patient was defined as having an abnormal capillaroscopic examination if more than 1 morphologic abnormality was present in at least 2 nail folds.

**Results:**

The median capillary density of TA patients was 9 (range: 9–11). There were no patients with capillary disorganization or avascular areas. Tortuous capillaries were detected in all patients. The other common morphological capillary abnormalities included enlarged/dilated capillaries (39.3%), branching capillaries (35.7%), and hemorrhages (32.1%). Only 1 patient had giant capillaries with early scleroderma-like pattern. Overall, there were 11 (39.3%) patients with abnormal capillaroscopic findings. There were more patients with abnormal capillaroscopic findings in the subgroup of TA patients whose upper extremity blood pressure could not be measured as compared to those whose blood pressure could be measured (66.7% vs. 26.3% patients; P = 0.04).

**Conclusion:**

Capillaroscopic abnormalities are frequently seen in TA patients. We consider that abnormal capillaroscopic findings in TA patients reflect the impaired blood flow due to narrowed or occluded arteries rather than the primary capillary involvement of the disease process.

## 1. Introduction

Takayasu arteritis (TA) is a large-vessel vasculitis mainly affecting the aorta and its branches [1]. Symptoms and/or signs due to small vessel involvement such as erythema nodosum or retinal vessel occlusion can also be encountered during the course of the disease [2,3]. Nail fold capillaroscopic examination is a simple method for detecting microvascular changes in the capillaries. It is mainly useful for the diagnosis of collagen vascular diseases, especially in patients suffering from Raynaud’s phenomenon due to systemic sclerosis [4]. The presence of capillaroscopic changes has also been reported in vasculitic syndromes [5]. The present study was conducted to define the capillaroscopic changes in TA. 

## 2. Materials and methods

TA patients who fulfilled the criteria for TA proposed by the American College of Rheumatology were enrolled in the study [6]. All subjects had a complete history and physical examination. Blood pressure measurements were performed from both upper extremities and recorded. Laboratory and imaging findings to determine the disease features were obtained from the hospital files. The type of vascular involvement was classified by using the Numano criteria [7]. 

Nail fold capillaroscopy (Videocapnet DS Medica, Milan, Italy) was performed in all subjects. Patients were at rest at room temperature (22–25 °C) for a minimum of 15 min before the examination, as defined previously [4]. The nail folds from the 2nd to 5th fingers (excluding the thumbs) on both hands were examined. Capillaroscopic examinations were evaluated in terms of capillary density, capillary architecture, and capillary morphology. The average value of the mean number of capillaries calculated from two fields (the number of capillaries in the distal row in 1 mm calculated from both sides in the middle of the nail fold divided by two) in each examined finger was used to determine the capillary density. Normal capillary density is considered as the presence of ≥9 capillaries per 1 mm [8]. The presence of elongated, tortuous, dilated, giant capillaries and hemorrhages were noted in the entire nail fold. Avascular areas were defined as loss of at least two consecutive capillaries in the dermal papilla. The presence of branching, bushy, or ramified capillaries was classified as neoangiogenesis. The percentage of tortuous and elongated capillaries was determined by the evaluation of the same fields that were used to determine capillary density [8,9]. To identify TA patients with abnormal capillary examination findings, we adapted the definitions suggested by Ingegnoli et al. [10]. A patient was defined as having an abnormal capillaroscopic examination if more than 1 morphologic abnormality (giant capillaries or >50% tortuous, >10% elongated capillaries, or hemorrhages or neoangiogenesis or avascular areas plus another capillaroscopic abnormality) was present in at least 2 nail folds. TA patients with scleroderma-like patterns were also noted.

All patients gave written informed consent. The study protocol was approved by the local research ethics committee.

### 2.1. Statistics

Statistical analysis was performed using SPSS 23.0 (IBM Corp., Armonk, NY, USA). Continuous data were described as mean (±standard deviation, SD) or median (interquartile range, IQR) and categorical variables as percentages. The chi-square test was used to compare categorical variables. Student’s t-test or the Mann–Whitney U test was used to compare continuous variables. P < 0.05 was considered significant.

## 3. Results

We studied 28 TA patients (27 females). The mean age and the median disease duration of the patients were 42.0 ± 14.8 years and 3 (±2.75) years, respectively. Vascular involvement was classified as type I in 8 (28.6%), type IIa in 3 (10.7%), type IIb in 5 (17.5%), type III in 1 (3.6%), and type V in 11 (39.3%) patients. Ten (35.7%) patients had hypertension (HT), 4 (14.3%) had diabetes mellitus (DM), and 2 (7.1%) had pulmonary hypertension (PH). All TA patients in this study were treated initially with steroids and immunosuppressive agents. At the time of capillaroscopic assessment, 23 (82.1%) were still on steroids and immunosuppressive or biologic agents. Three patients were using only steroids and 1 patient was followed without medication. 

**Figure 1:**
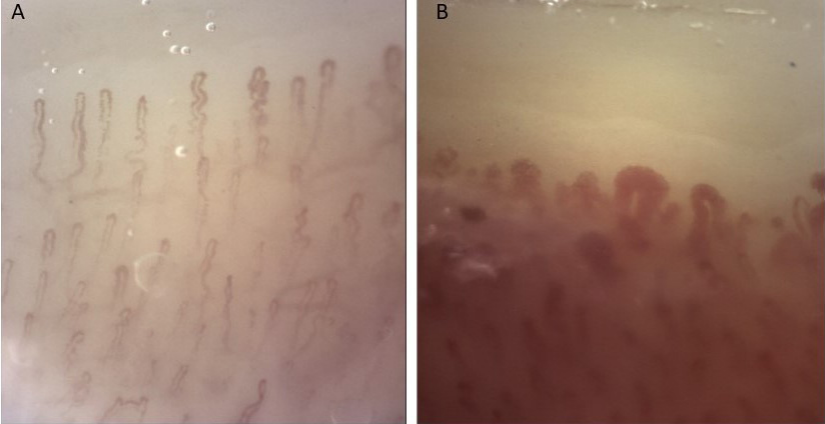
Nail fold capillaroscopy in Takayasu’s arteritis: A) tortuous capillaries, normal pattern; B) enlarged and giant capillaries, scleroderma-like pattern (original magnification 200×).

The median capillary density of TA patients was 9 (minimum: 9; maximum: 11). There were no patients with capillary disorganization. Tortuous capillaries were detected in all patients, but only 1 patient had >50% capillary tortuosity. The other common morphological capillary abnormalities were enlarged/dilated capillaries (39.3%), branching capillaries (35.7%), and hemorrhages (32.1%). Only 1 patient had giant capillaries. Capillaroscopic examination findings of the TA patients are summarized in the Table. The capillaroscopic images of the patients are shown in Figures 1A and 1B.

**Table:** Capillaroscopic examination findings of the TA patients.

Median capillary density (min–max)	9 (9–11)
Disorganization of capillary architecture, n (%)	0 (0)
Avascular area, n (%)	0 (0)
Enlarged/dilated capillaries, n (%)	11 (39.3)
Giant capillary, n (%)	1 (3.6)
Capillary tortuosity, n (%) ≤50% >50%	28 (100)27 (96.4)1 (3.6)
Elongated capillaries, n (%)≤10% >10%	6 (21.4)1 (3.6)5 (17.9)
Hemorrhages, n (%)	9 (32.1)
Neoangiogenesis, n (%)Branching capillariesBushy capillariesRamified capillaries	10 (35.7)10 (35.7)0 (0)0 (0)
Min: Minimum, max: maximum.	

There were 11 (39.3%) TA patients with abnormal capillaroscopic examination findings. Only 1 patient had scleroderma-like pattern (with giant capillaries: early scleroderma pattern). The mean age (43.7 ± 15.9 vs. 40.8 ± 14.5 years; P = 0.62) and the median disease duration (2 (8) years vs. 3 (1.5) years; P = 0.79) were not different between TA patients with and without abnormal capillaroscopic examination findings. The distribution of patients with abnormal capillaroscopic examination findings was not different with respect to TA vascular involvement types (P = 0.25). The percentage of patients with abnormal capillaroscopic examination findings was not different in TA patients with DM, HT, or PH as compared to patients without these conditions (38.5% vs 40.0%; P = 0.93). There were more patients with abnormal capillaroscopic findings in the subgroup of TA patients whose upper extremity blood pressures could not be measured as compared to the ones whose blood pressure could be measured (6/9 (66.7%) vs. 5/19 (26.3%) patients; P = 0.04).

## 4. Discussion

In this study, 39.3% of the TA patients had abnormal capillaroscopic examination findings. On the other hand, there was only one patient with a scleroderma-like pattern. We mostly observed mild capillaroscopic abnormalities; there were no patients with avascular areas or disorganization, and capillary density values were normal in all TA patients. TA patients whose blood pressures could not be measured from the upper extremities had more frequent abnormal capillaroscopic examination findings.

Capillaroscopic examination has become an increasingly used method for the differential diagnosis of Raynaud’s phenomenon and early diagnosis of systemic sclerosis [4]. The presence of capillaroscopic abnormalities and even scleroderma patterns have been reported in vasculitis [5]. Capillaroscopic examination findings have already been reported in primary vasculitis syndromes including Behçet’s disease, Henoch–Schönlein purpura, and Wegener’s granulomatosis [11–13]. In one study the presence of enlarged capillaries was found to be associated with lower age at disease onset, high blood pressure, and superficial phlebitis in Behçet’s disease, although another study did not confirm those findings [11,14]. Nail fold edema was suggested to be representative for disease activity in Henoch–Schönlein purpura [12]. The association between reported capillaroscopic abnormalities and clinical manifestations are yet to be defined. There are only limited data about capillaroscopic findings in TA. Javinani et al. investigated capillaroscopic findings in 15 TA patients and compared them with 15 healthy controls. They found that the capillary length and venous limb diameter were lower in TA patients as compared to controls. TA patients had more tortuous capillaries; on the other hand, there were no differences in capillary density between the groups [15]. In our study, capillary density was normal in all TA patients and no capillary disorganization was detected. Tortuous capillaries were detected in all patients, but only 1 patient had >50% capillary tortuosity. Other common morphological capillary abnormalities were dilated capillaries, branching capillaries, and hemorrhages. Only 1 patient had giant capillaries with an early scleroderma-like pattern. Overall, we observed 11 (39.3%) patients with abnormal capillaroscopic examination findings according to our predefined criteria, independently of age and disease duration. Our study results indicate the presence of mostly minor capillaroscopic abnormalities in TA patients, as in the previous one. Although most of our findings were consistent with the previous study, about 40% of our patients had enlarged or dilated capillaries, which seemed to diverge from the previous study’s findings. Dilated capillaries are among common capillaroscopic abnormalities that can be seen in up to 50% of healthy subjects [10]. We presented our results as percentages and did not give measurement results of our patients’ capillaries; this could be the cause of this difference. Also, differences in the mean age and the disease duration of the patients between studies may contribute to slightly different results. Decreased capillary density, enlarged and/or short capillaries, and capillary tortuosity can be seen in patients with DM and HT [8,16]. Nail fold capillary loss was also reported in patients with PH [17]. We did not find any difference in the number of TA patients with abnormal capillaroscopic examination findings who had DM, HT, or PH as compared to patients who did not have these conditions.

In this study, abnormal capillaroscopic findings were more frequent in patients whose blood pressures could not be measured from the upper extremities. Hypoperfusion and chronic hypoxemia of ocular tissue due to common carotid involvement are important mechanisms for the development of Takayasu’s retinopathy [3]. Similarly, impaired blood flow in the distal upper extremities may be the cause of abnormal capillaroscopic findings in TA patients. We did not demonstrate major capillaroscopic abnormalities such as capillary loss, avascular areas, or capillary disorganization in any TA patients. The presence of branched capillaries may reflect the repair phase of the damage due to impaired blood flow. Ramified and bushy capillaries, which represent defective angiogenesis, are typical findings in systemic sclerosis [4]. We consider that the absence of these bizarrely shaped capillaries along with mild capillaroscopic morphological abnormalities in TA patients suggests that capillary changes are likely secondary to impaired blood flow. Our results support the previous finding that TA patients with subclavian artery involvement had more prominent capillaroscopic abnormalities than the patients without subclavian artery involvement [15].

This study was conducted with a limited number of patients. All patients were treated with immunosuppressive agents and had a relatively long follow-up period. It is known that immunosuppressive agents can change the capillaroscopic findings in collagen tissue diseases [18,19]. As we studied a limited number of patients and there is no satisfactory method for defining the disease activity of TA, we did not assess the effect of disease activity on capillaroscopic changes, although some subtle abnormalities have been previously described [15]. Also, the small number of patients with DM and HT in our study population may have masked the changes in capillaries that can be seen in these conditions [8,16]. Therefore, capillaroscopic evaluation of untreated TA patients and long-term follow-up of their capillaroscopic findings are needed to demonstrate the clinical impact of the changes that we documented.

In conclusion, capillaroscopic abnormalities are frequently seen although mild in TA patients. We consider that abnormal capillaroscopic findings in TA patients reflect the impaired blood flow due to narrowed or occluded arteries rather than the primary capillary involvement of the disease process.
